# Teratogenic Effects of Sulfur Mustard on Mice Fetuses

**Published:** 2012

**Authors:** Nasrin Sanjarmoosavi, Naser Sanjarmoosavi, Marziyeh Shahsavan, Mohammad Hassanzadeh-Nazarabadi

**Affiliations:** 1 *Department of Medical Genetics, Faculty of Medicine, Mashhad University of Medical Sciences, Mashhad, Iran*; 2 *Department of Basic Sciences, Mashhad Azad University, Mashhad, Iran*

**Keywords:** Malformation, Sulfur Mustard, Teratogenicity

## Abstract

**Introduction:**

Sulfur Mustard (SM) has been used as a chemical warfare agent, in the World War I and more recently during Iraq-Iran war in early 1980s’. Its biological poisoning effect could be local or systemic and its effect depends on environmental conditions, exposed organs, and the extent and duration of exposure. It is considered as a strong alkylating agent with known mutagenic, carcinogenic effects; although a few studies have been performed on its teratogenicity so far.

**Materials and Methods:**

Mice were administered with SM intraperitoneally with a dose of 0.75 and 1.5 mg/kg in different periods of their gestation (gestational age of 11, 13 and 14 weeks). Control mice groups were included. Between 5 and 9 mice were used in each group. Dams underwent cesarean section on day 19 of their gestation. External examination was performed on the animals investigating craniofacial and septal defects and limb malformations such as adactyly and syndactyly. All data were analyzed by Chi-Square test and Fisher's exact test. The *P-* value less than 0.05 was considered significant.

**Results:**

Craniofacial and septal defects as well as the limb malformations were the most common types of birth defects, displaying an extremely complex biomedical problem.

**Conclusion:**

This study confirms a significant correlation between SM exposure and its teratogenic effect. We postulated that the malformations could be caused by an uncontrolled migration of neural crest cells, causing developmental disorders. In addition to environmental factors, modifying genes could play an important role in the pathogenesis of the defects.

## Introduction

Bis (2-chloroethyl) sulphide commonly known as sulphur mustard (SM) is a highly reactive bifunctional compound, categorized as an antimitotic, mutagenic, carcinogenic, teratogenic and cytotoxic agent (1). Sulphur mustard is a chemical warfare agent, which was largely used during the World War One and also in Iraq-Iran war. In World War І (1914-1918) the use of chemical weapons especially SM, caused the death of thousands (2-4). Although there are presently more toxic chemical warfare agents, mustard gas has remained the chemical weapon of choice in modern tactical warfare, as evidenced by its use during the Iran-Iraq war from 1983 to 1988 caused the death of hundreds of soldiers and civilians (2). 

The somatic damaging effects of SM have been well explained (5, 6). The eyes, the skin, and the respiratory tract are the main target organs of SM toxicity (7-9). SM is highly lipophilic and it is absorbed fast through the skin. Only, after a latent period of 6-24 hr, erythema and blisters appear on the skin of affected individuals (7). Pulmonary complications mainly on the upper respiratory tract such as hemorrhagic inflammation, cough, bronchitis, sore throat, hoarseness, and bronchopneumonia were observed in SM-exposed victims (7, 10). Besides, lung cancer has been reported in fisher men who were exposed to SM and in workers of SM manufacturing plants (7, 11-13). 

There are three distinct biochemical effects of SM: cytostaticity, cytotoxicity and mutagenicity (5). Although so much effort has been focused on understanding the mechanisms of direct cellular injury mediate by SM exposure, the cellular effect is still not known. Several mechanisms have been proposed for the cytotoxicity of SM such as DNA damage, labilization of lysosomes and calcium mediated toxicity (7, 8, 14, 15). It has been postulated that SM like other mustards such as nitrogen mustard could possess teratogenic effects (16). There is a few evidence of the effect of SM on development and reproduction. Reproduction abnormalities followed by chronic exposure to SM were observed by several workers, including foetus malformation and foetus mortality (15). For example craniofacial malformations are major human birth defects with a worldwide frequency of 700 and substantial clinical impacts (17-20). Facial clefts represent the majority of these defects and can arise at any stage of development due to perturbation. SM could alter the extracellular matrix as well as the migration, proliferation, and differentiation of cells (17). In the human fetus, the formation of limb occurs approximately 4 weeks after fertilization. The formation of the apical ectodermal ridge occurs at approximately 5 weeks and the digital radiation approximately 6 weeks after fertilization (21, 22). In the interdigital space, physiologic death of cells occurs, and the interdigital chasm is formed. If physiologic death of cells does not occur, simple syndactyly happens (22). Cardiovascular defects occur in almost 1% of newborns. Septation and alignment defects make up the largest single group of cardiac malformations. Most cases of congenital heart diseases are considered to be a result of complex interactions between environmental and genetic factors (23).

In the case of an SM attack during a war or a terrorist attack, pregnant women might be one of the victims who could survive the SM-exposure (3); however, the transplacentally exposed fetus could tolerate long term consequences of the poison. Since relatively a few studies has been conducted to assess the impact of SM on fetus teratogenicity, studying the SM teratogenic effects is essential. The aim of this study was to define the teratogenic effects of SM on mouse embryo.

## Materials and Methods

***Reagents***

Phenytoin (Dilantin® Desitiin Company, Humburg) and Propylene glycol (Merck Company, Germany) were purchased. SM was synthesized. All prepared solutions were stored at 4 °C in a dark place until administration. 

***Animals care statement***

The experiments were performed under the ethical guidance of Animal Care Regulations of Mashhad University of Medical Sciences. Eighty male and female NMRI mice were purchased from Razi Institute (Hesarak, Iran) and acclimatized for one week prior to treatment. Virgin NMRI albino mice, weighting 28-30 g (8-9 weeks old) were used as experimental and control mice in this study. The animals were maintained in a climate-controlled room under a 12 hr alternating light/dark cycle (9.00- 21.00 hr light), 20.1 to 21.2 ^°^C temperature and 50 to 55.5 % relative humidity. Throughout the experiment, they were housed in a specific pathogen-free facility on corncob bedding with dry food pellets and water was *ad libitum*. Female mice were randomly assigned to positive control, negative control and SM intraperitoneally injected –treated groups as shown in Table 1. Five to nine female mice were housed in each group (Table 1). Gestational Day (GD) was defined as the date on which the vaginal plug was observed.

***Animal treatment with SM***

Pregnant females were administered 0.75 and 1.5 mg /kg intraperitoneally in different days of gestation (GD 11, 13 and 14). These doses were applied with regard to LD_50_ of 4.4 mg/kg on GD 7; 32 the dose which could kill 50% of a group of animals under stated conditions. Control group was given the same concentration of phenytoin or propylene glycol. The schedule of administration is outlined in Table 1. On GD 19, the mice were euthanized by overdose of sodium thiopental. The gravid uterus of the pregnant mouse was harvested and weighed. The numbers and positions of the alive or dead fetuses, as well as reabsorptions were recorded.

***Fetal body weight and fetal assessment***

Following the euthanization of the mouse, an incision was made in the uterus and the umbilical cord connected to the fetus was cut; then each fetus was weighed (by using Sartorius PT210, Switzerland). Fetuses were categorized as alive or dead; their gender and their external abnormalities were examined. Normal palatogenesis was assessed based on microscopic examination of the palate surface after an incision was made through the temporal-mandibular joint. Cleft palate was scored if there was not fusion between the secondary palatal shelves. 

***Pathological analysis***

Pathological examination of the main organs (heart and kidney) was performed by stereo microscopy. 

***Statistical analysis***

The data was analyzed by Chi-Square test followed by Fisher's Exact Test. The *P-* value less than 0.05 was considered significant. The analysis was carried out using SPSS software (Version 16).

## Results

**Figure 1:**
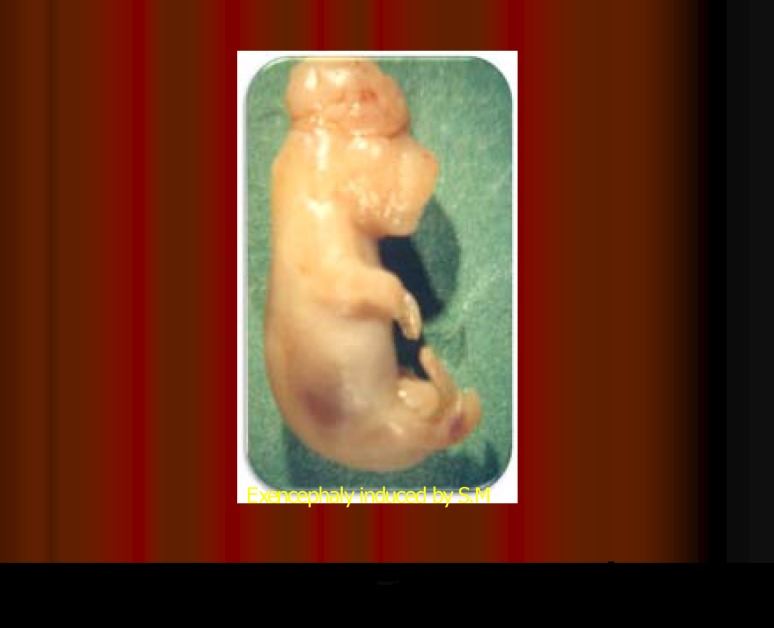
Exencephaly induced by sulfur mustard in mice

The effects of SM during pregnancy in experimental groups compared with control groups has been shown and summarized in Table 2. There were no indicative organ anomalies observed in the control group. These results showed that the incidence of cleft malformations in phenytoin treated group was higher than control groups. In addition, current data demonstrated that injection of 1.5 mg/kg of SM in GD 11 significantly increased the incidence of anomalies in comparison with the control group (*P*< 0.001), but there was no obvious teratogenic activity in GD14 SM-treated mice. A teratogenic effect of exencephaly induced by sulfur mustard is shown in Figure 1. The rate of anomalies was also slightly increased in GD11 compared with GD13. On the other hand, the incidence of malformations were more significant in mice receiving 1.5 mg/kg than 0.75 mg/kg of SM (*P*= 0.01). 

**Table 1:** Intraperitoneally injection schedule of different drugs with definition of fetuses and the frequency of anomalies. GD: Gestational day, CP: Cleft Palate, SD: Septal defect, ULE: Upper limb ectrodactyly, LLE: Lower limb ectrodactyly, ULS: Upper limb syndactyly, LLS: Lower limb syndactyly, SM: Sulphur mustard

No.			Used material		Number of pregnant mice	Day of injection		Injection dose			Injection volume		Live Fetus		Dead fetus		Resorbed fetus			CP	SD		ULE		LLE		ULS			LLS	
1			Phenytoin		9	G.D. 12			0.75 mg/kg		0.1 ml		77		0		0			34														
2		_		8			_			_		_		74		0		0	0																
3	Propylene glycol			8			G.D. 11			1.5 mg/Kg		0.1 ml		66		0		0	0													
4	Propylene Glycol			7			G.D. 13			1.5 mg/Kg		0.1 ml		69		0		0	0													
5	SM			5			G.D. 11			1.5 mg/Kg		0.1 ml		50		3		9	28			6		5		4		26	21			
6	SM			6			G.D. 13			1.5 mg/Kg		0.1 ml		59		0		4	21			2		4		6		7	5			
7	SM			7			G.D. 14			1.5 mg/Kg		0.1 ml		55		0		0	0			0		0		0		0	0			
8	SM			6			G.D. 11			0.75 mg/Kg		0.1 ml		62		1		0	12			0		2		13		11	0			
9	SM			5			G.D. 13			0.75 mg/Kg		0.1 ml		46		0		0	0			0		0		0		0	0			

**Table 2:** Definition of fetuses and the frequency of anomalies after intraperitoneally. injection of Sulphur mustard. GD:Gestational day, CP: Cleft palate, SD: Septal defect, ULE: Upper limb ectrodactyly, LLE: Lower limb ectrodactyly , ULS: Upper limb syndactyly, LIS: Lower limb syndactyly

Group	Injection dose	GD	Numbers of pregnant mice	Live fetus	Dead fetus	CP	SD	ULE	LIE	ULS	LIS
Group A	1.5 mg/kg	11	5	50	3	28	6	5	4	26	21
Group B	1.5 mg/kg	13	6	59	0	2	2	4	6	7	5
Group C	1.5 mg/kg	14	7	55	0	0	0	0	0	0	0
Group D	0.75 mg/kg	11	6	62	1	12	0	2	13	11	0
Group E	0.75 mg/kg	13	5	46	0	0	0	0	0	0	0
Total	--------	--------	29	272	4	42	8	11	23	44	26

**Figure 2:**
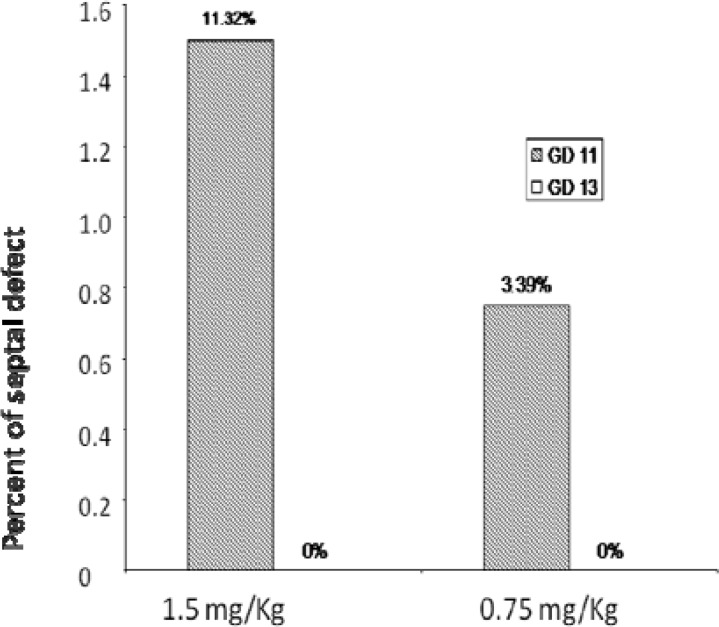
Rate of septal defect with different doses of Sulphur mustard in different days of gestation of mice

Pathological examination of the main organs (heart and kidney) was summarized in Figure 2.

## Discussion

Sulfur mustard (SM), commonly known as mustard gas, is an alkylating agent, which was widely used as a chemical warfare during Iran-Iraq war (2). Previous reports have demonstrated the ability of this class of compounds to cause adverse effects. However, very few correlations have been established between SM exposure and its teratogenic effects. Similar experimental studies were carried out on its analog; nitrogen mustard, which showed different malformations such as cleft palate, functional and structural anomalies and some growth defects (16). 

Our study showed that administration of SM in GD 11 can lead to more anomalies in contrast to GD 13. Thus, it indicates that the rate of congenital malformations increased during the period of organogenesis. Upon treatment with low dose of SM (0.75 mg/kg) there was no significant anomalies observed compared with the negative control group. Besides, no teratogenic effect of SM treatment was observed in GD 14.

These results suggested that teratogenic effects of SM were restrictively under the influence of a "threshold dose" and a "critical period" of gestation during organogenesis. Critical period for each organ or tissue existed i.e., an exposure to a single teratogen in a specific day might cause the anomaly. On the other hand, organ specific critical period may take several days long and the sensitivity of organs to teratogens could vary vastly in different days. Therefore, a specific dose of a teratogen in different days may cause different anomalies and increases the rate of malformations. In a similar study by McNamara *et al*, (29) pregnant rats were exposed to SM by gastric intubation in different doses. It was claimed that no evidence of teratogenicity was observed. Such a discrepancy could be explained by different route of drug administration and doses used in mice compare to rats.

Teratogens could affect different cells with several pathogenetic pathways such as mutation, cytotoxicity and enzymatic changes. A number of mechanisms have been proposed for these pathways including DNA damage, labialization of lysosomes and calcium mediated toxicity (7). Several studies have documented the mutagenic effects of SM in mammalian cells, in a wide variety of animal species and also *in* *vitro* test systems. The toxicity of SM has also been reviewed by Korkmaz and colleagues. They postulated that in addition to genetic mutations, epimutations can also involve in the pathogenesis of a variety of human diseases. They proposed that epigenetic aberrations may be responsible for delayed detrimental effects of mustard poisoning (24). Previously, it was found that bifunctional SM gives three major products of alkylation, of which two are monofunctional adducts and one cross linked adduct involving guanines on the same strand or complementary strands of DNA. The alkylated purines, 7-alkyl-G and 3-alkyl-A are unstable and are released spontaneously from SM-treated DNA at physiological pH and temperature by cleavage of the fl-glycosidic bond giving rise to apurinic sites (25). This has been related to the formation of DNA breaks leading to cytotoxicity of SM. Unfortunately, it is not clear how mustard gas causes severe multi-organ damage years after even a single exposure (26). The emphasis on teratogenic influences has not led to elucidation of pathogenetic pathways, so the potential mechanisms of induction of congenital defects by SM could be considered as important areas of research in future. The only relevant information in humans is that from Pour-Jafari (1992) (27), who reported an increased incidence of congenital malformations among offspring of Iranian chemical victims (males and females). In an oral study in animals, fetal toxicity was observed in reduced body weight and dysossification (28). The limited data available suggests that adverse developmental effects occur at the doses or exposure levels that could produce maternal toxicity. 

## Conclusion

This study indicated that within a population of pregnant mice, exposure to SM was directly correlated with increase risk of congenital malformations. Further studies are needed to study the molecular mechanisms involved in the teratogenesis from exposure to SM.
